# Cell-free miRNAs may indicate diagnosis and docetaxel sensitivity of tumor cells in malignant effusions

**DOI:** 10.1186/1471-2407-10-591

**Published:** 2010-10-28

**Authors:** Li Xie, Xi Chen, Lifeng Wang, Xiaoping Qian, Tingting Wang, Jia Wei, Lixia Yu, Yitao Ding, Chenyu Zhang, Baorui Liu

**Affiliations:** 1 The Comprehensive Cancer Center of Drum Tower Hospital Affiliated to Medical School of Nanjing University & Clinical Cancer Institute of Nanjing University, Zhongshan Road 321#, Nanjing 2l0008, PR China; 2 Jiangsu Diabetes Center, State Key Laboratory of Pharmaceutical Biotechnology, Life Science School of Nanjing University, Nanjing 2l0093, PR China; 3 Department of Hepatobiliary Surgery, Drum Tower Hospital Affiliated to Medical School of Nanjing University, Zhongshan Road 321#, Nanjing 210093, PR China

## Abstract

**Background:**

Circulating cell-free microRNAs have been identified as potential cancer biomarkers. However, the existence and the potential application of cell-free miRNAs in effusion samples are still uncertain. In order to explore the potential role of cell-free miRNA in malignant effusions, we selected 22 miRNAs differentially expressed in the serum of lung cancer patients and studied their expression levels in body cavity effusion samples.

**Methods:**

We measured the expression of 22 miRNAs using qRT-PCR in two samples, which were pooled with 18 malignant and 12 benign effusions, respectively. After discarding 9 lowly expressed miRNAs, a panel of 13 miRNAs were measured in 29 samples (benign n = 11, malignant n = 18). We also carried out a WST-8 test to evaluate the docetaxel sensitivity of tumor cells directly isolated from 15 malignant effusions.

**Results:**

We compared the miRNA expression levels between benign and malignant effusions using a Mann-Whitney U test and found miR-24, miR-26a and miR-30d were expressed differently between the two groups (*P *= 0.006, 0.021 and 0.011, respectively). Cells isolated from effusions rich in cell-free miR-152 were more sensitive to docetaxel (r = 0.60, *P *= 0.016).

**Conclusions:**

Collectively, our study demonstrated that cell-free miRNAs in the supernatant of effusions may aid in the diagnosis of malignancy and predict chemosensitivity to docetaxel.

## Background

Body cavity effusion is a clinical common manifestation and may cause dilemmas in treatment. Diagnosis of effusions is of particular importance for cancer patients and a co-existing malignant effusion implies an advanced stage of tumor and intricate clinical management. Currently, diagnosis of malignant effusions mainly relies on cytological analysis. However, the sensitivity is still limited to about 70% even with repeated analyses [1]. Thus, alternative diagnostic methods are still in need to assist in the diagnosis of effusions. The supernatant of effusion samples may contain useful information in the form of proteins and nucleic acids. Tumor marker proteins [2], DNA methylation status [3] and cell-free mRNA levels [4] have been identified as potential biomarkers with limited improvement in diagnostic accuracy.

MicroRNAs (miRNAs) are a group of short RNAs that post-transcriptionally regulate expression of proteins by binding to the 3'UTRs of target mRNAs. miRNAs are involved in the development of cancer and specific miRNA expression profile may suggest not only the disease status but also the prognosis and response to chemotherapeutic reagents [5,6]. Accumulating evidence suggested circulating cell-free miRNAs as potential biomarkers for disease status, such as liver injury [7], diabetes [8], myocardial infarction [9] and more importantly, cancer [8,10]. Specific circulating miRNA profiles may act as novel biomarkers for cancer diagnosis and early detection. Thus, we hypothesized that malignant effusions may have a specific cell-free miRNA profile, which convey malignant information regarding the presence of tumor cells. We chose a panel of miRNAs that were deregulated in the serum of lung cancer patients and explored their possible role in malignant effusions. We also correlated the level of cell-free miRNA with sensitivity of tumor cells against docetaxel, a microtubule stabilizer widely used in cancer management.

## Methods

### Samples collection and Patients Information

Our study was approved by the Ethics Review Board of Nanjing University, Nanjing, China. Informed consent was obtained from all patients. All effusion samples were recruited from the Affiliated Drum Tower Hospital, Medical School of Nanjing University, from Jan 2006 to Dec 2007. Malignant effusions were confirmed by pathological diagnosis. Samples were collected before chemotherapy. Effusions were diagnosed as benign based on the clinical context and the absence of malignant cells in 3 separate samples from the same patients. No cancers occurred in patients with benign effusions over the next 6 months. All samples were transported to the laboratory within 30 min after their collection. The information for patients is presented in Table 1.

**Table 1:** Patient characteristics.

	Characteristics	Number	Percent (%)
Malignant		18	
	Gastric cancer	9	50
	Lung cancer	9	50
	Gender Male	9	50
	Female	9	50
	Age < 60	7	39
	> 60	11	61
Benign		11	
	Ascites	4	36
	Pleural	7	64
	Gender Male	6	55
	Female	5	45
	Age < 60	6	56
	> 60	5	45
	Diagnosis		
	Liver Cirrhosis	2	18
	Tuberculosis	3	27
	Heart Failure	3	27
	Pneumonia	2	18
	Injury	1	9

### Chemotherapeutic agent and chemosensitivity test

Primary tumor cells were prepared by Ficoll-Hypaque (specific gravity 1.077, Pharmacia) density centrifugation. The number and viability of cells were determined by cytological examination with trypan blue dye. The test drug concentration of docetaxel (DOC, donated by Jiangsu Hengrui Medicine Company, purity > 99.9%) was 12.4 μM, derived from pharmacokinetic data and adjusted for protein-binding to approximate the concentration achievable in the patient plasma. Cells were seeded in U-shape-96-well plates at 5× 10^4 ^cells/well and treated with drugs in triplicate for 72 h. WST-8 reagent (10 μl each) from Cell Counting Kit-8 (Dojindo, Kumamoto, Japan) was added and absorbance was determined with a multiwell spectrophotometer (BioTek, VT, USA) at 460 nm. Inhibition rate = (1-absorbance of treated cells/control cells) ×100%. Only the specimens containing at least 50% cancer cells were enrolled for chemosensitivity test. And we have proved the increase of tumor cells number by the culture in our preliminary test. Serum free medium and polypropylene U-shape-96-well microplates can enrich tumor cells up to 80-90% [11].

### Quantitative RT-PCR (qRT-PCR) analysis of miRNA expression

Samples for miRNA quantification were centrifuged at 8000 g for 20 min and the supernatants were collected. After adding 750 μl Trizol LS (Invitrogen, CA, USA) into 250 μl effusion samples, another 25 μl ath-miR156a (20 nM, synthesized by Takara, Japan) was supplemented into each sample tube. Total RNA was extracted using Trizol LS reagent (Invitrogen, CA, USA) according to the manufacture's instruction. The miRNA expression levels were quantified by real-time PCR using miRNA detection kit (Applied Biosystems, Foster city, CA, USA). Briefly, miRNAs were reverse-transcribed to cDNA with the AMV reverse transcriptase (Takara, Japan). Subsequently, real-time PCR was performed on Mx3000P cycler (Stratagene, USA). All reactions were run in triplicate. Ct data were determined using default threshold settings. The relative expression levels of miRNAs were calculated as 2^-ΔΔCt ^[12], using ath-miR156a as reference and a mixture of RNA extracted from human lung cancer A549 cells and gastric cancer BGC-823 cells (1:1) as the calibrator.

### Statistics

Mann-Whitney U test was used to compare the expression of each miRNA between malignant and benign groups. Spearman correlation coefficient was used to analyze the relationship between miRNA levels and inhibition rates of docetaxel on tumor cells. All these statistics were done by a statistics program, SPSS version 13.1 (SPSS Inc, Chicago, IL, USA). A *P *value < 0.05 (two tailed) was considered statistically significant.

## Results

### The stability of miRNAs in the effusion samples

**Figure 1:**
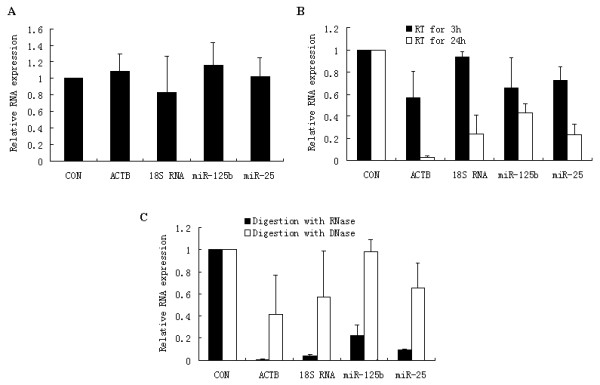
**Stability of cell-free RNAs in body cavity effusion samples**. Effusion samples (n = 3) was subjected to 10 freeze-thaw cycles (A), room temperature conservation for 3 h or 24 h (B) and RNase or DNase digestion for 3 h at 37°C (C). Total RNA was extracted from the effusion samples. The levels of miRNA or other RNAs were determined using qRT-PCR with 40 cycles. Column, relative expression levels of RNA compared to samples without treatment; bars, SD; ACTB: β-actin.

We firstly tested the stability of cell-free miRNAs in the effusion samples kept at room temperature for different time periods, or treated with multiple freezing and thawing, and digestion with RNase or DNase. β-actin mRNA transcripts and 18S RNA representing large molecular RNAs were analyzed in this study. qRT-PCR was carried out to quantify the RNAs of three samples with various treatments. Multiple freeze-thaw cycles had hardly any effect on cell-free RNAs in effusion samples (Figure 1A). When effusion samples were left at room temperature for 3 h or 24 h, cell-free RNAs began to degrade at 3 h. β-actin mRNA degraded quickly and significantly, while 18S RNA and the two miRNAs were more stable, with more than 20% remaining after 24 h (Figure 1B). When treated with RNase, miRNAs were more stable than large RNA molecules although significant deposition also occurred in miRNAs. All cell-free RNAs were resistant to DNase digestion (Figure 1C).

### Expression levels of cell-free miRNAs in malignant and benign effusion samples

In order to evaluate the miRNAs expression in the supernatant of effusion samples, we selected 22 miRNAs from the list of deregulated cell-free miRNAs in the serum of lung cancer patients published previously [8]. qRT-PCR was used to examine the possible expression levels in two samples, which were pooled from 18 malignant and 12 benign effusions (equivalent amounts of RNA were added to each sample). Ct values of each miRNAs were obtained and assessed. miRNAs with Ct >35 were discarded, resulting in 13 miRNAs (Table 2).

**Table 2:** Initial evaluation of expression levels for 22 miRNAs.

miRNA	Ct value	
	Sample B	Sample M
**miR-20a**	29.85	27.74
**miR-21**	30	26.62
miR-22	35.63	33.1
**miR-24**	33.06	29.35
**miR-25**	33.17	31.75
**miR-26a**	30.96	27.91
**miR-26b**	33.26	31.69
miR-27a	35.96	33.24
miR-27b	35.47	33.74
**miR-29a**	34.11	34.58
**miR-30d**	34.52	30.15
miR-145	35.91	36.51
**miR-146a**	28.61	28.8
**miR-152**	33.95	31.75
miR-199a	No Ct	37.9
miR-200c	35.31	34.15
miR-221	36.05	34.25
**miR-222**	29.87	27.46
**miR-223**	29.3	27.45
**miR-320**	25	23.28
miR-375	No Ct	No Ct
miR-382	No Ct	No Ct

Sample B: the benign sample pooled from 5 ascites and 7 pleural effusions

Sample M: the malignant sample pooled from 9 ascites and 9 pleural effusions

**Figure 2:**
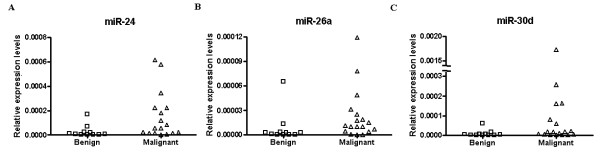
**Expression levels of miR-24, miR-26a and miR-30d in effusion samples**. (A) The expression levels of miR-24 were higher in malignant effusions (n = 18) than benign effusions (n = 11 *P *= 0.006); (B) The expression levels of miR-26a were higher in malignant effusions (n = 18) than benign effusions (n = 11, *P *= 0.021); (C) The expression levels of miR-30d were higher in malignant effusions (n = 18) than benign effusions (n = 11, *P *= 0.011).

Next, we measured the expression levels of 13 miRNAs in 29 samples (benign n = 11; malignant n = 18). The expression levels of the 13 miRNAs were compared between malignant and benign groups using a Mann-Whitney U test. miR-24, miR-26a and miR-30d were expressed significantly differently between groups (*P *= 0.006, 0.021 and 0.011, respectively). All three miRNAs were significantly over-expressed in the supernatant of malignant effusions (Figure 2). However, no significant difference was identified after Bonferroni correction for the three miRNAs differently expressed between malignant and benign groups.

### Cell-free miRNAs in effusion samples may predict cell sensitivity against docetaxel

**Figure 3:**
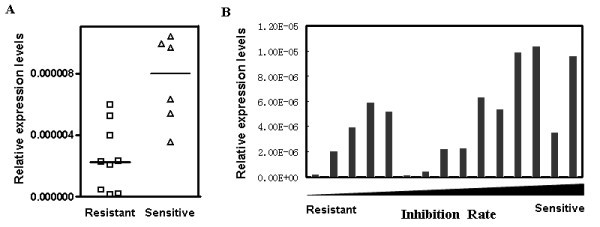
**Relationship between expression levels of miR-152 and docetaxel sensitivity of tumor cells**. (A) Relative expression levels of cell miR-152 in docetaxel resistant and sensitive samples; (B) Spearman correlation analysis showed that miR-152 may correlate to the docetaxel sensitivity in 15 effusion samples (Spearman correlation coefficient r = 0.60, *P *= 0.016).

The WST-8 test has been confirmed to be a reliable chemosensitivity test for primary tumor cells of malignant effusions [13]. In the current study, the chemosensitivity of primary tumor cells isolated from fifteen malignant effusion samples were examined. The mean inhibition rate was 0.49 (range: 0.2 to 0.86, n = 15). Malignant effusion samples with an inhibition rate < mean were considered docetaxel resistant, and those with an inhibition rate ≥ mean were considered as docetaxel sensitive. The docetaxel sensitive group has a higher level of cell-free miR-152 in the effusion supernatant (*P *= 0.005, Figure 3A). Spearman correlation coefficient was also used to compare the miRNAs expression levels and sensitivity. The expression levels of miR-152 correlated with the inhibition rates of docetaxel (r = 0.60, *P *= 0.016, Figure 3B). miR-320 expression levels were also associated with docetaxel inhibition rates (r = 0.52, *P *= 0.042), but no significant difference on miR-320 expression levels was observed between docetaxel resistant and sensitive groups (*P *= 0.087).

## Discussion

In the current study, we explored the expression of cell-free miRNAs in malignant effusion samples, and found that malignant effusions have higher expression levels of miR-24, miR-26a and miR-30d. This suggests that malignant effusions may have a different cell-free miRNA expression profile. miR-24, miR-26a and miR-30d are differentially expressed in a panel of cancers [14-17]. Chen et al [8] demonstrated that these three miRNAs were highly expressed in the serum of lung cancer patients compared to healthy control. Our result is in accordance with the result in the serum of lung cancer patients.

This study also revealed that effusion samples with tumor cells resistant to docetaxel contain lower levels of cell-free miR-152 than those from chemo-sensitive tumor cells. We demonstrated for the first time that cell-free miRNAs may be potential diagnostic biomarkers for diagnosis and drug sensitivity.

Currently used biomarkers mainly rely on tumor specific peptides derived from proteomic profiling [18]. However, as tumor-associated proteins constitute only a minor fraction compared with normal proteins, the sensitivity for these "tumor proteins" may be limited. Cell-free nucleic acids have recently attracted interest as the diagnostic biomarkers for cancer. Several studies have focused on cell-free nucleic acids from fluid samples and explored their potential application as diagnostic biomarkers [4], [19]. Up to now, cell-free miRNAs have been studied well in plasma, serum, urine [20], saliva [21] and sputum [22]. Recently published reports proved that cell-free miRNAs in plasma or serum are stable and can be used to distinguish cancer patients from healthy subjects [8,10,23]. In view of the success of cell-free miRNAs of other body fluids in cancer diagnosis and the results of this study, cell-free miRNAs may serve as novel diagnostic biomarkers in the diagnosis of body cavity effusions with minimal invasiveness and sample requirement.

Docetaxel is a tubulin-binding agent that induces cell death through stabilizing microtubules after binding to β-tubulin. Interogation of miRGen, a web tool for miRNA target prediction and function [24], revealed a group of microtubule related genes that may be potential targets of miR-152, including β- tubulin2b, β-tubulin 4q-chain, β-tubulin 6 and β-tubulin 8. A group of ATP-binding cassettes (ABC) transporters was also identified as potential targets of miR-152, including ABCA1, ABCB7 and ABCD3. Chemotherapeutic resistance of tumor cells may be due to the drug pumps, which are mainly made up of ABC proteins [25]. These two types of possible targets may explain partly the reason for lower expression of cell-free miR-152 in docetaxel resistant group. However, data is still scarce and further research is needed.

## Conclusions

In summary, we showed that malignant effusion supernatant had a different profile of miRNAs when compared to benign samples. The expression levels of cell-free miR-152 may discriminate docetaxel sensitive samples from resistant samples. Given the results from this study, additional potential markers may be revealed by more systematic miRNA profiling in the future.

## Competing interests

The authors declare that they have no competing interests.

## Authors' contributions

LX carried out the miRNA quantification and drafted the manuscript. XC developed the detection protocol. TW, XQ and LW collected samples and carried out drug sensitivity assay. JW made the statistical analysis. LY and YD participated in data analysis and helped to draft the manuscript. BL and CZ designed and planed the experiment and drafted the manuscript. All authors read and approved the final manuscript.

## Pre-publication history

The pre-publication history for this paper can be accessed here:

http://www.biomedcentral.com/1471-2407/10/591/prepub
